# Alterations in the MicroRNA of the Blood of Autism Spectrum Disorder Patients: Effects on Epigenetic Regulation and Potential Biomarkers

**DOI:** 10.3390/bs8080075

**Published:** 2018-08-15

**Authors:** Tamara da Silva Vaccaro, Julia Medeiros Sorrentino, Sócrates Salvador, Tiago Veit, Diogo Onofre Souza, Roberto Farina de Almeida

**Affiliations:** 1 Institute of Health’s Basic Science, Department of Biochemistry, Federal University of Rio Grande do Sul, Porto Alegre 90035-000, RS, Brazil; tamara.genetica@gmail.com (T.d.S.V.); juliamsorrentino@gmail.com (J.M.S.); diogo.bioq@gmail.com (D.O.S.); 2 Pediatric Neurology Center, Porto Alegre Clinical Hospital (HCPA), Federal University of Rio Grande do Sul, Porto Alegre 90035-903, RS, Brazil; socratessalvador@gmail.com; 3 Institute of Health’s Basic Science, Department of Microbiology, Immunology and Parasitology, Federal University of Rio Grande do Sul, Porto Alegre 90035-190, RS, Brazil; tiagoveit@terra.com.br

**Keywords:** ASD, microRNA, SIRT1, HDAC2, PI3K/Akt-TSC:mTOR, MeCP2

## Abstract

**Aims:** Autism spectrum disorder (ASD) refers to a group of heterogeneous brain-based neurodevelopmental disorders with different levels of symptom severity. Given the challenges, the clinical diagnosis of ASD is based on information gained from interviews with patients’ parents. The heterogeneous pathogenesis of this disorder appears to be driven by genetic and environmental interactions, which also plays a vital role in predisposing individuals to ASD with different commitment levels. In recent years, it has been proposed that epigenetic modifications directly contribute to the pathogenesis of several neurodevelopmental disorders, such as ASD. The microRNAs (miRNAs) comprises a species of short noncoding RNA that regulate gene expression post-transcriptionally and have an essential functional role in the brain, particularly in neuronal plasticity and neuronal development, and could be involved in ASD pathophysiology. The aim of this study is to evaluate the expression of blood miRNA in correlation with clinical findings in patients with ASD, and to find possible biomarkers for the disorder. **Results:** From a total of 26 miRNA studied, seven were significantly altered in ASD patients, when compared to the control group: miR34c-5p, miR92a-2-5p, miR-145-5p and miR199a-5p were up-regulated and miR27a-3p, miR19-b-1-5p and miR193a-5p were down-regulated in ASD patients. **Discussion:** The main targets of these miRNAs are involved in immunological developmental, immune response and protein synthesis at transcriptional and translational levels. The up-regulation of both miR-199a-5p and miR92a-2a and down-regulation of miR-193a and miR-27a was observed in AD patients, and may in turn affect the SIRT1, HDAC2, and PI3K/Akt-TSC:mTOR signaling pathways. Furthermore, MeCP2 is a target of miR-199a-5p, and is involved in Rett Syndrome (RTT), which possibly explains the autistic phenotype in male patients with this syndrome.

## 1. Introduction

Autism spectrum disorder (ASD) refers to a group of heterogeneous brain-based neurodevelopmental disorders with different levels of symptom severity in two core domains: impairment in communication and social interaction; and repetitive and stereotypic patterns of behavior [1]. Given the challenges, the clinical diagnosis of ASD is based on information gained from interviews with patients’ parents, in accordance with the Diagnostic and Statistical Manual of Mental Disorders, Fifth Edition, Text Revision (DSMV-TR). In April 2018 an important study from the Autism and Developmental Disabilities Monitoring Network was published by the U.S. Department of Health and Human Services/Centers for Disease Control that estimates the prevalence of ASD among U.S. children aged 8 years old. The results obtained revealed that one in 59 children aged 8 years, from multiple communities, presented ASD [1]. Multiple lines of evidence suggest that ASD is genetic in origin, with most data supporting a polygenic model [2,3]. However, except for the GWAS study [4], which demonstrate that although autism possesses a complex genetic architecture, common variations are found, and other genetic studies have been quite successful in identifying suitable candidate genes for ASD. The heterogeneous pathogenesis of this disorder appears to be driven by genetic and environmental interactions, which also play a vital role in predisposing individuals to ASD with different commitment levels [5]. In recent years, it has been proposed that epigenetic modifications directly contribute to the pathogenesis of several neurodevelopmental disorders, such as ASD [6,7,8]. Epigenetics modifiers act at the interface of genes targeting different mechanisms: histone modifications, DNA methylation, chromatin remodeling or noncoding RNA (microRNAs); controlling heritable changes in gene expression without changing the DNA sequence [9,10].

It has been proposed that epigenetic machinery was closely related with neuronal development disorders by several pathways influencing cell cycle regulation, development and axon guidance, dendritic spine development and function, actin cytoskeleton regulation and protein synthesis regulation [11]. The microRNAs (miRNAs) comprise a species of short noncoding RNA (approximately 21 nucleotides) that regulate gene expression post-transcriptionally, by interacting with specific mRNAs, usually at the 3′ untranslated region (UTR), through partial sequence complementation, resulting in mRNA degradation or repression of translation [12]. In this way, a variety of cellular processes could be affected, as cellular differentiation, metabolism and apoptosis [13]. miRNAs have an essential functional role in the brain, particularly in neuronal plasticity and neuronal development [14]. Each miRNA binds multiple targets in several mature mRNA species, mediating several biological functions, including physiological neuronal gene expression and also several pathological processes, as were previously described in some brain disorders [15,16]. The expression of many miRNAs is dynamically regulated during brain development, neurogenesis, the neuronal maturation mechanism [14], and an important signaling pathway involved in all of these processes is the tuberous sclerosis complex-mammalian target of rapamycin (TSC-mTOR). For example, a significant impairment to this pathway has been observed in some disorders related to autism, including Tuberous Sclerosis, a disease characterized by mutations in tuberous sclerosis proteins (TSC) TSC1 or TSC2 genes [17].

Recently, studies have revealed associations between the immune system and the central nervous system in ASD development, hypothesizing that early neuroimmune disturbances during embryogenesis could persist throughout an individual’s lifetime. Considering that miRNAs can pass into the bloodstream from cells or tissues and organs [18], it seems reasonable to suppose that changes in miRNA levels in blood could reflect direct changes occurred in the Central Nervous System and lymphoid organs. Thus, in the present study, we evaluated the expression of miRNA in the blood of ASD patients and analyzed the repression targets of these miRNAs, correlating them with biochemical pathways that may be deregulated in ASD. We propose that the miRNA expression profile may be used as a clinical marker for the diagnosis or prognosis of the disorder, and that epigenetic changes may help in understanding the disease.

## 2. Materials and Methods

### 2.1. Subjects

This study was approved by the Ethics Committee of Hospital de Clínicas de Porto Alegre (HCPA) and the subjects’ parents provided informed consent before inclusion in the study. Eleven patients attending an outpatient clinic of the HCPA were included in the study, following a semi-structured interview. Clinical diagnosis was confirmed by criteria defined by the Diagnostic and Statistical Manual of Mental Disorders V and Autism Screening Questionnaire. Seven ASD male patients with a mean age of 7.5 years (sd 2.5) and four non-ASD male controls with a mean age of 7.5 years (sd 2.5) was carried out. The ASD subjects were enrolled in the same autism severity group by two well-validated clinical tests: the Childhood Autism Rating Scale (CARS) [19] and Autism Diagnostic Observation Schedule-Generic (ADOS-G) [20]. Exclusion criteria were as follows: (a) comorbidities such as chromosomal syndromes; (b) genetic or metabolic disease. Non-ASD male children were included after clinical diagnosis that excluded: (a) presence of psychiatric disorder; (b) presence of ASD patients in family; (c) presence of chromosomal syndrome; (d) genetic or metabolic disease.

### 2.2. Quantitative Real Time Polymerase Chain Reaction (RT-qPCR)

MicroRNA profile evaluations were evaluated from peripheral blood samples were obtained from patient and immediately mixed with a three-fold volume of Trizol (Invitrogen) for total RNA extraction.

Considering some common aspects among the different disorders that affect the Central Nervous System, the 26 candidate miRNAs evaluated in this study, were based in previous studies in the literature conducted with patients with sleep disturbances [21] or who had some neurodegenerative diseases [22,23].

miRNA quantification was carried out by quantitative RT-qPCR using a stem-loop RT-PCR technique, as previously described [24]. Complementary DNA (cDNA) was synthesized from mature miRNA using a reverse transcriptase reaction containing 2 µg of total RNA, 1 µL of 10 mM dNTP mix (Invitrogen, Waltham, MA, USA), 3 µL of stem loop RT primer mix, 4 µL M-MLV reverse transcriptase 5X reaction buffer (Invitrogen), 2 µL of 0.1 M DTT (Invitrogen), 1 µL of RNase inhibitor (Invitrogen), 1.0 µL of M-MLV reverse transcriptase (Invitrogen), and sterile distilled water to a final volume of 20 µL. The synthesis of cDNA was completed after a sequence of four incubations at 16 °C for 30 min, 42 °C for 30 min, 85 °C for 5 min and 4 °C for 10 min.

The quantitative PCR mix was made up with 12 µL of cDNA (1:33), 1.0 µL of specific miRNA forward and universal reverse (10 µM) primers (as detailed in Table 1), 0.5 µL of 10 µM dNTP mix, 2.5 µL of 10X PCR buffer (Invitrogen), 1.5 µL of 50 mM MgCl2 (Invitrogen), 2.4 µL of 1X Sybr Green (Molecular Probes, Eugene, OR, USA), 0.1 µL of Platinum Taq DNA Polymerase (Invitrogen), and sterile distilled water to a final volume of 24 µL. The fluorescence of Sybr Green was used to detect amplification, estimate Ct values, and to determine specificity after melting curve analysis. PCR cycling conditions were standardized to 95 °C for 5 min, followed by 40 cycles at 95 °C for 10 s, 60 °C for 10 s, and 72 °C for 10 s. After the main amplification, sample fluorescence was measured from 60 °C to 95 °C, with an increasing ramp of 0.3 °C each, in order to obtain the denaturing curve of the amplified products to assure their homogeneity after peak detection, and Tm (melting temperature) estimation using data obtained from an Applied Biosystems StepOne System (Lincoln Centre Drive Foster City, CA, USA). After the main amplification step, sample fluorescence was measured at temperatures from 55–99 °C, with an increasing ramp of 0.1 °C, in order to obtain the denaturing curve of the amplified products and to assure their homogeneity after peak detection and Tm estimation using data obtained from Applied Biosystems StepOne Plus.

The relative expression was obtained in triplicate using the 2^−Δ∆Ct^ method, where the Crossing threshold (Ct) values of the target samples are subtracted from the average Ct values of the standard or control samples. The use of 2^−Δ∆Ct^ is adequate, as the amount of RNA among the different blood samples to produce the cDNAs did not differ significantly and produced similar Ct values among the samples for 4 different miRNA used in the initial screening and evaluated by Genorm software. The Genorm analysis was used to assess the variance in the expression levels among the miRNA and pairwise comparisons. This resulted in 4 control miRNA, the most stable ones, for every pairwise comparison, serving as normalizers to evaluate the relative expression of miRNA. The RT-qPCR results were analyzed by Genorm algorithm to assess the variance in expression levels of the miRNA studied. This program performed a scan of the present miRNA from groups compared two by two each time. Then, the expression stabilities of the set of miRNA were evaluated. All miRNA were ranked according to their stability value. A pairwise variation analysis was performed by Genorm to determine the number of miRNA required for accurate normalization and to identify which miRNA could be used as internal control.

### 2.3. Statistical Analysis

To assess whether ASD patients and controls were homogeneous for age and gender the Chi-squared test was used. Statistical analysis of the relative expression values obtained for each miRNA in the experimental group was performed by Student’s *t*-test implemented using the SPSS Statistics 17 software. In order to compare the expression levels between the two experimental groups, the Waller–Duncan and Tukey HSD tests were performed with SPSS 17, with identical group discrimination and similar probability values.

Each altered miRNA has a cluster of validated targets, which were predicted for at least two available programs for experimental assays (miRBase).

The protein clusters were formed by the STRING 10 software using an automatic force-directed layout algorithm that orders the nodes in the network. The algorithm works iteratively trying to position the nodes apart from each other with a “preferred distance” proportional to the String global score (https://string-db.org/). All nucleotide sequences were screened and evaluated through miRBase and, after the ready profile, miRBase data banks were searched for altered microRNA targets (www.mirbase.org).

## 3. Results

**Figure 1:**
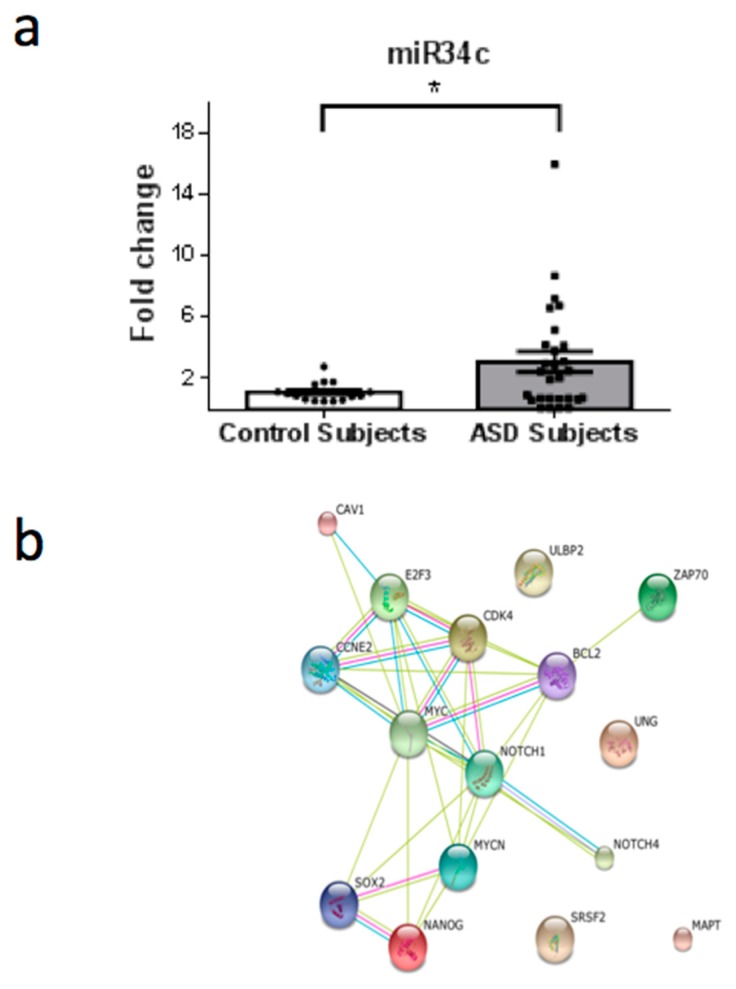
(**a**) Scatter plot of differential relative expression of miR34c in peripheral blood of ASD subjects compared to control subjects. Results expressed as mean ± standard error. *t*-Test analysis; * *p* < 0.05. (**b**) mRNA validates targets for miR34c selected in miRBase and respective proteins clusters that can be involved in ASD. Proteins involved in epigenetic regulation: NANOG (Nanog homeobox); NOTCH1 (notch 1); SOX2 (SRY (sex determining region Y)-box 2); SRSF2 (serine/arginine-rich splicing factor 2); NOTCH4 (notch 4); E2F3 (E2F transcription factor); MYCN (v-myc myelocytomatosis viral related oncogene, neuroblastoma derived); MYC (v-myc myelocytomatosis viral oncogene homolog). Proteins involved in cell cycle: CCNE2 (cyclin E2); BCL2 (B-cell CLL/lymphoma 2). Proteins involved in immunological regulation: ZAP70 (zeta-chain (TCR) associated protein kinase 70kDa); ULBP2 (UL16 binding protein 2); CDK4 (cyclin-dependent kinase 4); CAV1 (caveolin 1). Protein associated with cytoskeleton stabilization in neuronal cell: MAPT (microtubule-associated protein tau (776 aa)). Protein involved in DNA repair: UNG (uracil-DNA glycosylase).

**Figure 2:**
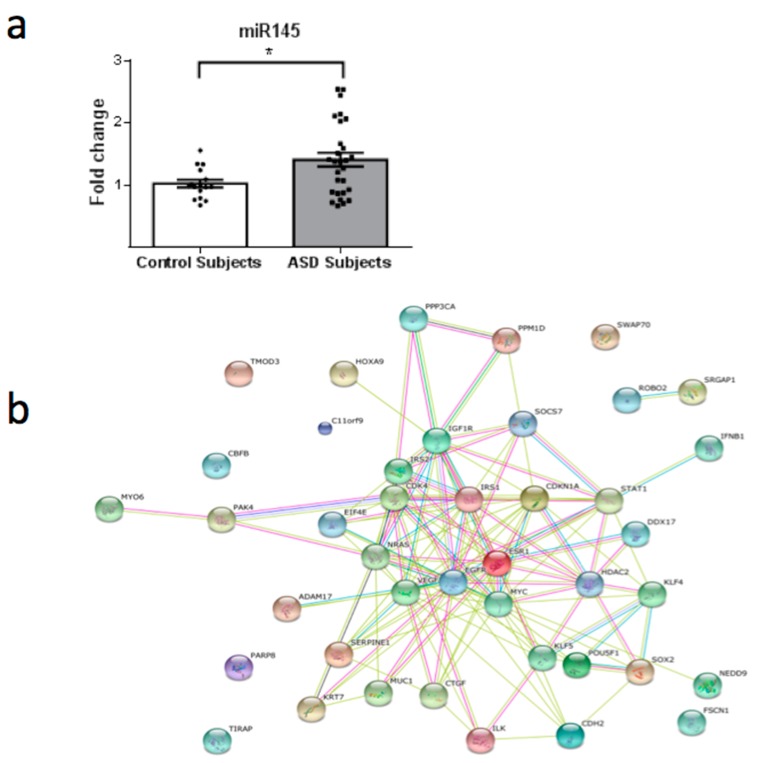
(**a**) Scatter plot of differential relative expression of miR145 in peripheral blood of ASD subjects compared to control subjects. Results expressed as mean ± standard error. *t*-Test analysis; * *p* < 0.05. (**b**) mRNA validates targets for miR145 selected in miRBase and respective proteins clusters that can be involved in ASD. Proteins involved in epigenetic regulation: ERS1 (estrogen receptor 1); POU5F1 (POU class 5 homeobox 1 (360 aa)); C11orf9 (chromosome 11 open reading frame 9); PARP8 (poly (ADP-ribose) polymerase family, member 8 (854 aa)); SOX2 (SRY (sex determining region Y)-box 2); HOX9 (homeobox A9); STAT1 (signal transducer and activator of transcription 1); KLF4 (Kruppel-like factor 4); KLF5 (Kruppel-like factor 5); NEDD9 (neural precursor cell expressed); DDX17 (DEAD (Asp-Glu-Ala-Asp) box helicase 17); EIF4E (eukaryotic translation initiation factor 4E); CBFB (core-binding factor, beta subunit); HDAC2 (histone deacetylase 2). Proteins involved in cell cycle: CDKN1A (cyclin-dependent kinase inhibitor 1A (p21, Cip1)); CDK4 (cyclin-dependent kinase 4); MYC (v-myc myelocytomatosis viral oncogene homolog); PPM1D (protein phosphatase, Mg^2+^/Mn^2+^ dependent, 1D); KRT7 (keratin 7). Proteins involved in immunological regulation: IFNB1 (interferon beta 1 fibroblast); TIRAP (toll-interleukin 1 receptor (TIR) domain containing adaptor protein); SOCS7 (suppressor of cytokine signaling 7); ADAM17 (ADAM metallopeptidase domain 17). Proteins involved in insulin metabolism: IGF1R (insulin-like growth factor 1 receptor), IRS1 (insulin receptor substrate 1); IRS2 (insulin receptor substrate 2). Proteins associated with cytoskeleton and cell migration: SWAP70 (SWAP switching B-cell complex 70kDa subunit); ILK (integrin-linked kinase); MYO6 (myosin VI); FSCN1 (fascin homolog 1, actin-bundling protein); ROBO2 (roundabout, axon guidance receptor, homolog 2); CDH2 (cadherin 2, type 1, N-cadherin (neuronal)), TMOD3 (tropomodulin 3 (ubiquitous)); SRGAP1 (SLIT-ROBO Rho GTPase activating protein 1); PAK4 (p21 protein (Cdc42/Rac)-activated kinase 4). Others: SERPINE1 (serpin peptidase inhibitor, clade E (nexin, plasminogen activator inhibitor type 1)); EGFR (epidermal growth factor receptor); NRAS (neuroblastoma RAS viral (v-ras) oncogene homolog); VEGFA (vascular endothelial growth factor A); PPP3CA (protein phosphatase 3, catalytic subunit); CTGF (connective tissue growth factor); MUC (mucin 1).

**Figure 3:**
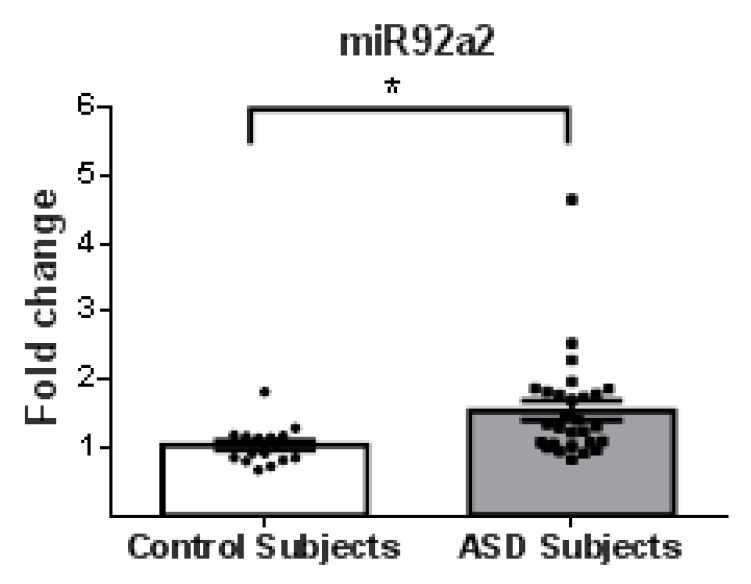
Scatter plot of differential relative expression of miR92a2 in peripheral blood of ASD subjects compared to control subjects. Results expressed as mean ± standard error. *t*-Test analysis; * *p* < 0.05. miR92a2 does not have validated targets in Homo sapiens.

**Figure 4:**
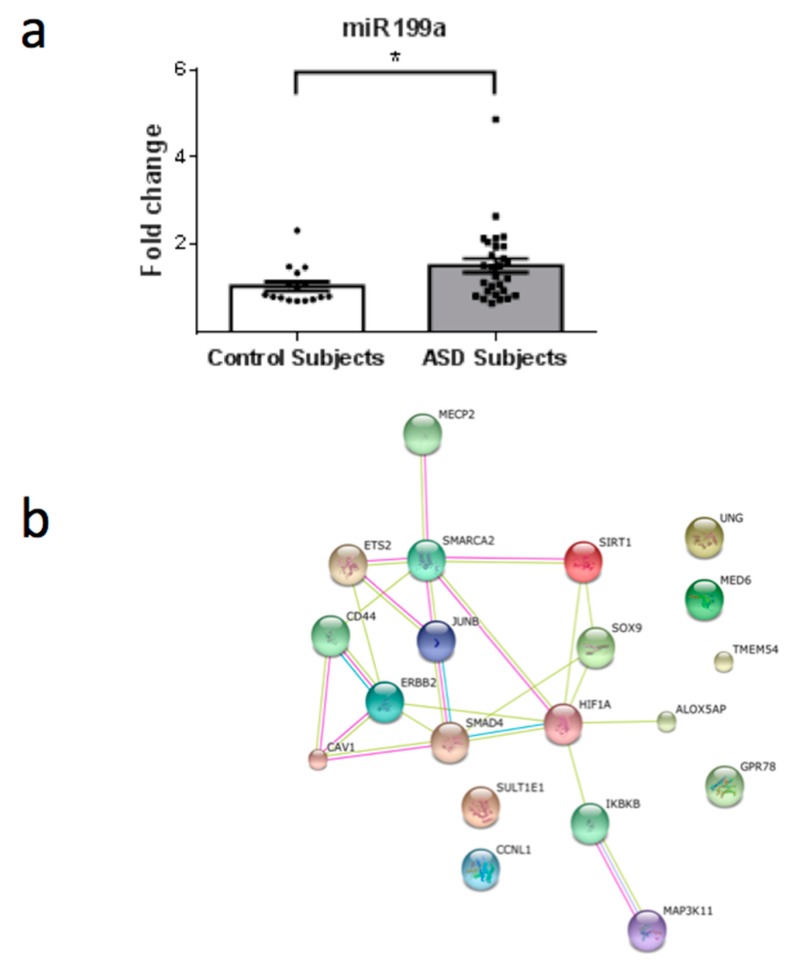
(**a**) Scatter plot of differential relative expression of miR199a in peripheral blood of ASD subjects compared to control subjects. Results expressed as mean ± standard error. *t*-Test analysis; * *p* < 0.05. (**b**) mRNA validates targets for miR199a selected in miRBase and respective proteins clusters that can be involved in ASD. Proteins involved in epigenetic regulation: SIRT1 (sirtuin 1); SOX9 (SRY (sex determining region Y)-box 9); MED6 (mediator complex subunit 6); SMARCA2 (SWI/SNF related, matrix associated, actin dependent regulator of chromatin, subfamily a, member 2); CCNL1 (cyclin L1); JUNB (jun B proto-oncogene); HIF1A (hypoxia inducible factor 1, alpha subunit); ETS2 (v-ets erythroblastosis virus E26 oncogene homolog 2); MECP2 (methyl-CpG binding protein 2). Proteins involved in immunological regulation: CAV1 (caveolin 1); SMAD4 (SMAD family member 4); ALOX5AP (arachidonate 5-lipoxygenase-activating protein); CD44 (CD44 molecule); IKBKB (inhibitor of kappa light polypeptide gene enhancer in B-cells). Protein associated with cytoskeleton and cell migration: ERBB2 (v-erb-b2 erythroblastic leukemia viral oncogene homolog 2). Protein involved in DNA repair: UNG (uracil-DNA glycosylase). Protein involved in strogen metabolim: SULT1E1 (sulfotransferase family 1E, estrogen-preferring, member 1). Others: MAP3K11 (mitogen-activated protein kinase kinase kinase 11); TMEM54 (transmembrane protein 54); GPR78 (G protein-coupled receptor 78; Orphan receptor).

**Figure 5:**
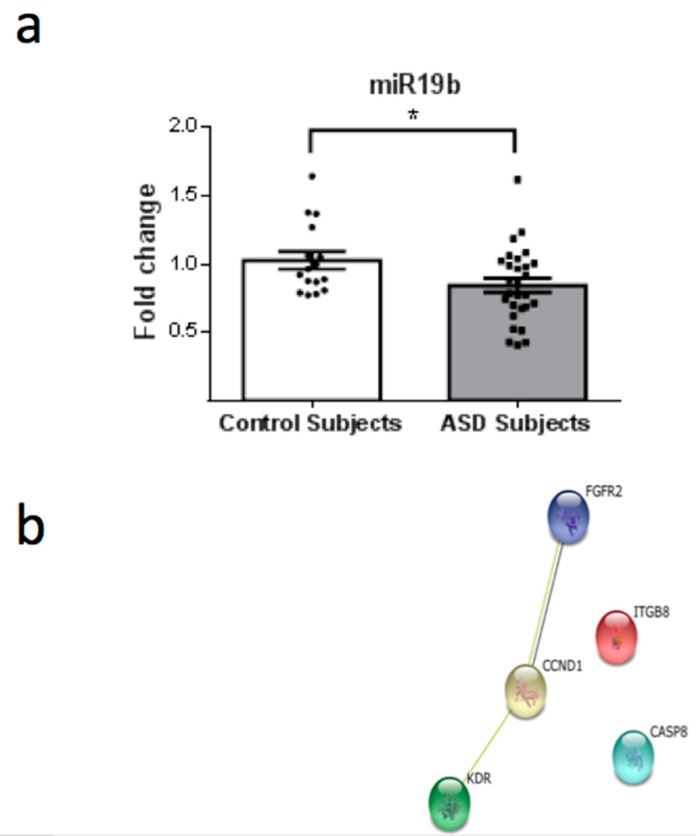
(**a**) Scatter plot of differential relative expression of miR19b in peripheral blood of ASD subjects compared to control subjects. Results expressed as mean ± standard error. *t*-Test analysis; * *p* < 0.05. (**b**) mRNA validates targets for miR19b selected in miRBase and respective proteins clusters that can be involved in ASD. Protein involved in cell cycle: CCND1 (cyclin D1). Proteins associated with cytoskeleton: ITGB8 (integrin, beta 8); KDR (kinase insert domain receptor). Others: CASP8 (caspase 8); FGFR2 (fibroblast growth factor receptor 2).

**Figure 6:**
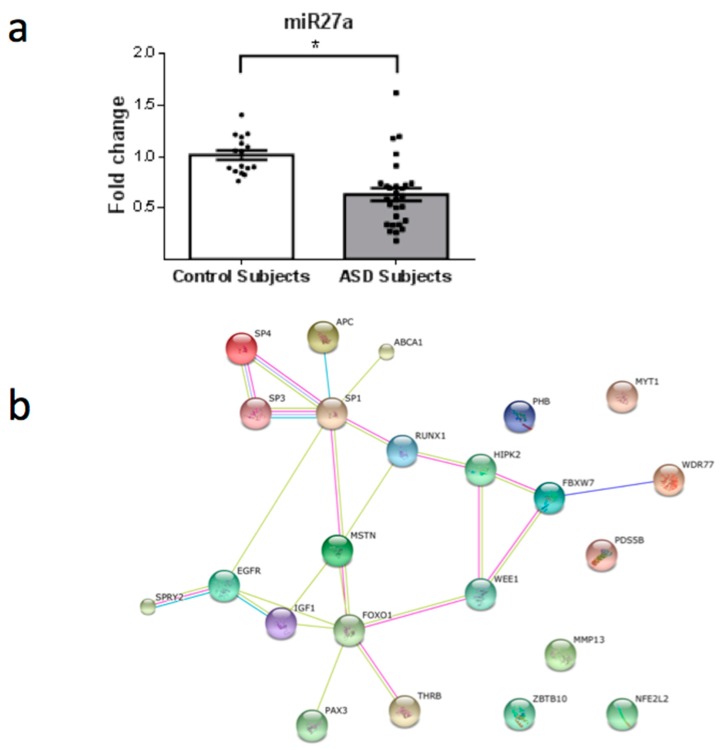
(**a**) Scatter plot of differential relative expression of miR27a in peripheral blood of ASD subjects compared to control subjects. Results expressed as mean ± standard error. *t*-Test analysis; * *p* < 0.05. (**b**) mRNA validates targets for miR27a selected in miRBase and respective proteins clusters that can be involved in ASD. Proteins involved in epigenetic regulation: SP4 (Sp4 transcription factor); SP3 (Sp3 transcription factor); WDR77 (WD repeat domain 77); RUNX1 (runt-related transcription factor 1); MYT1 (myelin transcription factor 1); SP1 (Sp1 transcription factor); FOXO1 (forkhead box O1); PAX3 (paired box 3); NFE2L2 (nuclear factor (erythroid-derived 2)-like 2); HIPK2 (homeodomain interacting protein kinase 2); ZBTB10 (zinc finger and BTB domain containing 10). Proteins involved in cell cycle: PHB (prohibitin), WEE1 (WEE1 homolog). Others: APC (adenomatous polyposis coli), MMP13 (matrix metallopeptidase 13 (collagenase 3)); MSTN (myostatin); EGFR (epidermal growth factor receptor); FBXW7 (F-box and WD repeat domain containing 7); IGF1 (insulin-like growth factor 1); PDS5B (regulator of cohesion maintenance, homolog B); THRB (thyroid hormone receptor, beta); ABCA1 (ATP-binding cassette, sub-family A (ABC1), member 1); SPRY2 (sprouty homolog 2).

**Figure 7:**
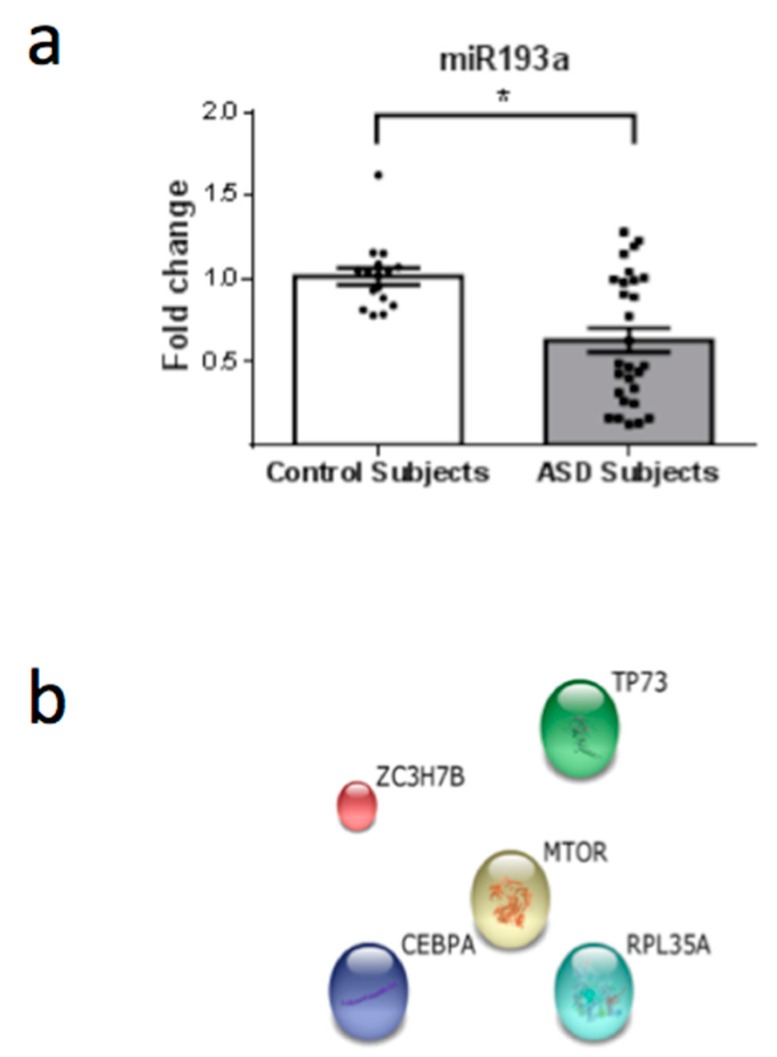
(**a**) Scatter plot of differential relative expression of miR193a in peripheral blood of ASD subjects compared to control subjects. Results expressed as mean ± standard error. *t*-Test analysis; * *p* < 0.05. (**b**) mRNA validates targets for miR193a selected in miRBase and respective proteins clusters that can be involved in ASD. Protein associated with central regulation of cellular metabolism, growth and survival in response to hormones, growth factors, nutrients, energy and stress signals: MTOR (mechanistic target of rapamycin (serine/threonine kinase). Protein involved in cell cycle: TP73 (tumor protein p73). Others: ZC3H7B (zinc finger CCCH-type containing 7B); RPL35A (ribosomal protein L35a); CEBPA (CCAAT/enhancer binding protein (C/EBP), alpha).

From a total of 26 miRNAs evaluated (Table 1), seven were statistically altered in ASD patients in comparison with control group. Specifically, miR34c-5p (Figure 1; *p* = 0.0068), miR-145-5p (Figure 2; *p* = 0.0099), miR92a-2-5p (Figure 3; *p* = 0.0026) and miR199a-5p (Figure 4; *p* = 0.047) were up-regulated, while miR19b-1-5p (Figure 5; *p* = 0.0184), miR27a-3p (Figure 6; *p* = 0.0001) and miR193a-5p (Figure 7; *p* = 0.001) were down-regulated comparing the ASD patients with the control group. Additionally, the validated targets of the seven altered miRNA are shown in protein clusters (Figures 1b–7b), except for the miR92a-2-5p, which does not have validated targets in *Homo sapiens*. The results for Genorm revealed ubiquitous and stably expressed normalization by RT-qPCR: miR125a-5p, miR181b-5p, miR125b-2-3p, miR198. Thus, each alerted miRNA was calculated from 4 normalizing miRNA.

Each altered miRNA has a cluster of validated targets, which were predicted for at least two available programs for experimental assays (miRBase). Using the STRING 10 software and information from validated targets for each miRNA available at the miRBase website (mirbase.org), we predicted the pathways that may be involved in ASD.

## 4. Discussion

ASD is characterized by tremendous phenotypic heterogeneity and the individuals affected demonstrate different levels of behavioral commitment. Considering the differences among ASD individuals, our study aimed to evaluate only one level in the autism spectrum, since the samples tested were only boys that had been clinically diagnosed with classical autism as well established by CARS [19] and (ADOS-G) [20]. As ASD has no biological marker and its etiology is unknown, our research may open new perspectives for investigating cellular processes that occur in affected individuals. The understanding of epigenetic alterations, such as miRNA modifications, could help in elucidate some relevant aspects of ASD, especially in classic autism. Immunological differences could explain the neuro-immunoregulation observed in ASD patients and based in the results shown here, we identified targets that could potentially be affected by altered miRNAs using the miRBase database. The influence of decreased levels of miRNA in ASD patients should be discussed given the fact that the miRNA targets identified are involved in several biological functions that have been previously reported to be dysregulated in ASD, such as cell cycle regulation, axon development and guidance, dendritic spine development and function, protein synthesis regulation and immune response. In addition, it should be taken into account that the intracellular targets for these altered miRNA might also be altered in autistic patients and that this data opens an interesting area of investigation, particularly in blood cell signaling and profiling. Besides the possibility of using this set of miRNA biomarkers for the diagnosis and prognosis of ASD, it is important to understand the pathways that are involved in the regulation of those molecules. Immune aberrations consistent with dysregulated immune responses have been reported in autistic children, including skewed TH1/TH2 cytokine profiles [25], low natural killer (NK) cell activity [26] and an imbalance in serum immunoglobulin levels [27]. In addition, ASD has been linked to autoimmunity and chronic neuroinflammation caused by glutamatergic excitotoxicity and diminished GABAergic signals [28].

The majority of the target proteins of miR34c-5p (Figure 1b)—which is up-regulated in ASD patients—are involved in cell cycle control, such as MYC and MYB. The most important target of miR34c-5p is ZAP70 (Zeta-chain-associated protein kinase 70), a signaling protein involved in immunological development and response. ZAP70 is one of the main proteins involved in lymphocyte activation and function and is also important for NK activity [29]. Additionally, cell-mediated immunity was impaired in ASD patients, as observed by low numbers of CD4^+^ cells and a concomitant T-cell polarity with an imbalance of Th1/Th2 subsets towards Th2 and the presence of autoantibodies against brain proteins [30]. Inhibition of ZAP70 protein expression could modify the adaptive response and the development of thymocytes. In a study involving 1027 blood samples from autistic children, 45% of a subgroup of children with autism suffered from low NK cell activity [27]. In another study, the cytotoxicity of NK cells was significantly reduced in ASD, as compared with controls [31]. Furthermore, under similar conditions, the presence of perforin, granzyme B, and interferon-gama (IFN-γ) in NK cells from ASD children was significantly lower in comparison to controls [31]. Similarly, it is sustained that lower levels in CD57+CD3− lymphocyte, a subset of NK cells, could be a possible link to a subgroup of ASD [26]. These findings suggest a possible dysfunction of NK cells in children with ASD. Therefore, during critical periods of development, abnormalities in NK cells could represent a susceptibility factor in ASD, that predispose to the development of autoimmunity and/or adverse neuroimmune interactions as already reported [31].

Furthermore, ASD is a neurodevelopmental disorder associated with abnormal neuroplasticity in early development [32]. Another target of miR34c-5p (Figure 1b) is a protein associated with cytoskeleton stabilization in neuronal cell: MAPT (microtubule-associated protein tau with 776 amino acids). The mRNA that encodes MAPT is regulated by alternative splicing, giving rise to several mRNA species [33]. MAPT transcripts are differentially expressed in the nervous system, depending on the stage of neuronal maturation and neuron type [34]. Additionally, mutations in this gene are also associated with neurodegenerative disorders, such as Alzheimer’s disease, Pick’s disease, frontotemporal dementia, cortico-basal degeneration and progressive supranuclear palsy [35]. Our findings suggest that by epigenetic modifications a negative regulation occurs in the MAPT protein, since changes in neuronal maturation have been widely reported in patients with ASD [33,34]. With regard to epigenetic regulation, the recruitment of histone acetyltransferases (HATs) and histone deacetylases (HDACs) is considered a key element in the dynamic regulation of many genes playing important roles in cellular proliferation and differentiation. The recruitment of HDACs leads to transcriptional repression and inhibitors of this enzymatic activity could reverse aberrant repression and lead to re-expression of genes inducing cell differentiation. In consonance with this hypothesis, the use of Valproic Acid (VPA), a widely used antiepileptic drug, which induces proteasomal degradation of HDAC2 and also inhibits selectively the catalytic activity of class I HDACs [36], during pregnancy is a risk factor associated with the increased incidence of ASD [37]. In this study, we observed that miR145-5p (Figure 2) is up-regulated in ASD patients and could promote mRNA degradation of HDAC2, thus inducing suppression of protein synthesis, since HDAC2 is a validated target of miR145-5p. Therefore, an autistic phenotype could be linked to HDAC2 deregulation due to mir145 up-regulation in our patients.

**Figure 8:**
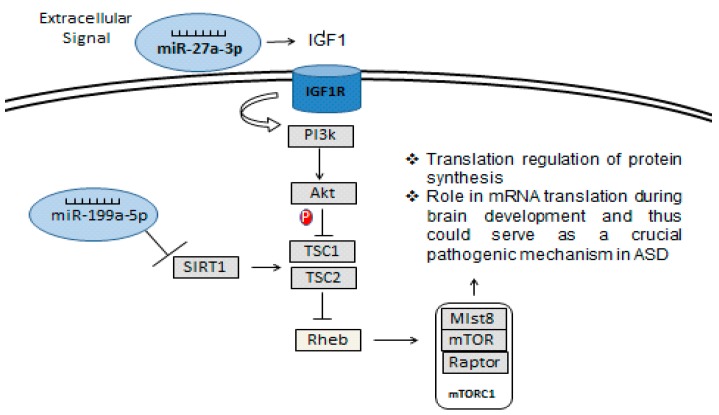
miRNA regulatory pathway in the control of the protein synthesis through SIRT1—TSC:mTOR.

Considering that SIRT1 enzyme is a NAD^+^ dependent deacetylase involved in a wide range of cellular processes, it was already shown that SIRT1 negatively regulates the mTOR (mammalian target of rapamycin) signaling, potentially through the TSC1/2 complex [38]. The TSC1/2-mTOR signaling pathway is reported to have a crucial role in mRNA translation during brain development and thus could serve as a crucial pathogenic mechanism in ASD. Furthermore, the up-regulation of miR-199a-5p (Figure 4) may induce a down-regulation of SIRT1 in ASD patients (Figure 8). Additionally, mTOR is a target of miR-193a, another miRNA found to be down-regulated in our study (Figure 7a). TSC1 is a predictive target of miR92a-2a (Figure 3), which was up-regulated in our ASD patients, possibly demonstrating and supporting epigenetic regulation in ASD patients, due to alterations in miRNA in this pathway.

On the other hand, this study demonstrated that the expression of miR-27a-3p (Figure 6) was significantly decreased in ASD patients. Therefore, its mRNA targets could reduce the repression of mRNA translation. An example of a target of miR-27a-3p is insulin-like growth factor-1 (IGF-1), which acts through IGF-1 receptor (IGF-1R, a tyrosine-kinase receptor) [39] and plays a pivotal role in several cellular responses (Figure 8). IGF-1 is a pleiotropic signal in the developing cerebellum, regulating proliferation, neurite outgrowth and survival [40]. The activated IGF-1R activates several phosphorylation cascades being one of the most commonly, the phospho-tidylinositol-3-kinase (PI3K) pathway. PI3K can induce activation of phosphokinase B/Akt (Akt), leading to inhibition of glycogen synthase-kinase-3-beta (GSK3β), and could promote proliferation and survival [41]. The Akt-mTOR pathway is dysregulated in multiple animal models of monogenic causes of ASD, including fragile X mental retardation [42], Rett Syndrome [43] and tuberous sclerosis [44], whereas IGF-1 ligands may improve neurodevelopmental symptoms in Rett Syndrome [45]. In addition, our results show down-regulation of miR-27a (Figure 6) in ASD patients and this could facilitate PI3K activation, indicating that the PI3K/Akt pathway could be up-regulated, which could be involved in the cellular growth, proliferation, migration and adhesion [46].

Remarkably, we found that MeCP2 (methyl-CpG binding protein 2), which is involved in Rett Syndrome (RTT), is a target of miR-199a. RTT is an X-linked postnatal neurodevelopmental disorder, which is primarily caused by mutations in the gene encoding MeCP2 [47]. A number of MeCP2 target genes have been identified, including the Brain-Derived Neurotrophic Factor (BDNF) [48]. Several RTT mutations have been described that render MeCP2 incapable of binding to methylated DNA and/or repressing gene transcription. This protein dysfunction has also been shown to cause abnormalities in RNA splicing, suggesting a complex molecular pathogenesis [49]. The loss of the function of the MeCP2 protein leads to autistic behaviors in RTT syndrome. As such, as MeCP2 is a known target of a miRNA found to be altered in our patients with classic autism, we suggest that the MeCP2 protein is decreased by epigenetic inhibition, leading to autistic behavior similar to that observed in patients affected by a mutation in the MeCP2 gene.

## 5. Conclusions

Despite the intense investigation of the genetic factors involved in ASD, there is still no consensus as to the main causative candidate genes. Therefore, studies of epigenetic regulation could identify altered mechanisms and elucidate the etiology of this disorder. This study shows changes in miRNA levels in patients that could explain many phenotypic features in the spectrum. The investigation of the modulation of proteins that regulate gene expression seems to be a correct path to follow, since the heterogeneity of the disease is an important factor and could be explained by the different levels of regulation. Although the small number of subjects enrolled in our study limits our data, we believe that the results presented here are innovative and important to increase the knowledge in the ASD field. Furthermore, it is important to highlight that the ASD subjects presented the same autism severity, and considering the accuracy and strength of the methodology used, this study produce important hypotheses that should be better explored in future studies. The alterations surveyed in the blood can be seen as permanent changes and correlate from the embryonic development of affected individuals. In addition, the altered miRNA set found in this study may be analyzed in a future molecular diagnosis of the disorder, since the alterations found corroborate with clinical and biochemical situations. The quantification of altered miRNA expression may be used for the prognosis of ASD, as scales of impairment may be made, since this disorder presents a spectrum of severity levels.

## Figures and Tables

**Table 1:** Information about miRNA evaluated. ID, Chromosome localization, Accession miRBase (www.mirbase.org), Mature Sequence and Forward Primer.

ID	Chromosome	Accession miRBase	Mature Sequence	Forward Primer
hsa-miR-19b-1-5p	13	MI0000074	AGUUUUGCAGGUUUGCAUCCAGC	AGTTTTGCAGGTTTGCATCCAGC
hsa-miR-24-2-5p	19	MI0000081	UGCCUACUGAGCUGAAACACAG	TGCCTACTGAGCTGAAACACAG
hsa-miR-25-3p	7	MI0000082	CAUUGCACUUGUCUCGGUCUGA	CATTGCACTTGTCTCGGTCTGA
hsa-miR-27a-3p	19	MI0000085	UUCACAGUGGCUAAGUUCCGC	TTCACAGTGGCTAAGTTCCGC
hsa-miR-29b-2-5p	1	MI0000107	CUGGUUUCACAUGGUGGCUUAG	CTGGTTTCACATGGTGGCTTAG
hsa-miR-31-5p	9	MI0000089	AGGCAAGAUGCUGGCAUAGCU	AGGCAAGATGCTGGCATAGCT
hsa-miR-34a-5p	1	MI0000268	UGGCAGUGUCUUAGCUGGUUGU	TGGCAGTGTCTTAGCTGGTTGT
hsa-miR-34c-5p	11	MI0000743	AGGCAGUGUAGUUAGCUGAUUGC	AGGCAGTGTAGTTAGCTGATTGC
hsa-miR-92a-2-5p	X	MI0000094	GGGUGGGGAUUUGUUGCAUUAC	GGGTGGGGATTTGTTGCATTAC
hsa-miR-99a-5p	21	MI0000101	AACCCGUAGAUCCGAUCUUGUG	AACCCGTAGATCCGATCTTGTG
hsa-miR-125a-5p	19	MI0000469	UCCCUGAGACCCUUUAACCUGUGA	TCCCTGAGACCCTTTAACCTGTGA
hsa-miR-125b-1-3p	11	MI0000446	ACGGGUUAGGCUCUUGGGAGCU	ACGGGTTAGGCTCTTGGGAGCT
hsa-miR-125b-2-3p	21	MI0000470	UCACAAGUCAGGCUCUUGGGAC	TCACAAGTCAGGCTCTTGGGAC
hsa-miR-145-5p	5	MI0000461	GUCCAGUUUUCCCAGGAAUCCCU	GTCCAGTTTTCCCAGGAATCCCT
hsa-miR-181b-5p	1	MI0000270	AACAUUCAUUGCUGUCGGUGGGU	AACATTCAUUGCTGTCGGTGGGT
hsa-miR-191-5p	3	MI0000465	CAACGGAAUCCCAAAAGCAGCUG	CAACGGAATCCCAAAAGCAGCTG
hsa-miR-193a-5p	17	MI0000487	UGGGUCUUUGCGGGCGAGAUGA	TGGGTCTTTGCGGGCGAGATGA
hsa-miR-193b-3p	16	MI0003137	AACUGGCCCUCAAAGUCCCGCU	AACTGGCCCTCAAAGTCCCGCT
hsa-miR-198	3	MI0000240	GGUCCAGAGGGGAGAUAGGUUC	GGTCCAGAGGGGAGATAGGTTC
hsa-miR-199a-5p	19	MI0000242	CCCAGUGUUCAGACUACCUGUUC	CCCAGTGTTCAGACTACCTGTTC
hsa-miR-210-3p	11	MI0000286	CUGUGCGUGUGACAGCGGCUGA	CTGTGCGTGTGACAGCGGCTGA
hsa-miR-214-3p	1	MI0000290	ACAGCAGGCACAGACAGGCAGU	ACAGCAGGCACAGACAGGCAGT
hsa-miR-221-3p	X	MI0000298	AGCUACAUUGUCUGCUGGGUUUC	AGCTACATTGTCTGCTGGGTTTC
hsa-miR-222-3p	X	MI0000299	AGCUACAUCUGGCUACUGGGU	AGCTACATCTGGCTACTGGGT
hsa-miR-339-5p	7	MI0000815	UCCCUGUCCUCCAGGAGCUCACG	TCCCTGTCCTCCAGGAGCTCACG
hsa-miR-370-3p	14	MI0000778	GCCUGCUGGGGUGGAACCUGGU	GCCTGCTGGGGTGGAACCTGGT
